# Central and Local Modulators of Reproduction and Fertility: An Update

**DOI:** 10.3390/ijms23095285

**Published:** 2022-05-09

**Authors:** Rosaria Meccariello

**Affiliations:** Department of Movement Sciences and Wellness, University of Naples Parthenope, Via Medina 40, 80133 Naples, Italy; rosaria.meccariello@unparthenope.it; Tel.: +39-081-5474-668

Infertility is currently one of the most important health troubles in industrialised countries after cardio-vascular diseases and cancer [1]. According to the World Health Organization, “Infertility affects millions of people of reproductive age worldwide—and has an impact on their families and communities. Estimates suggest that between 48 million couples and 186 million individuals live with infertility globally” [1]. Hence, the need to broaden the knowledge on the modulators of reproduction and fertility in both sexes, in order to expand the plethora of possible markers and therapeutic targets in the clinical setting. 

Reproduction and fertility depend on the activity of the hypothalamus–pituitary–gonad (HPG) axis and are highly sensitive to environmental factors such as diet, stress or endocrine disrupting chemicals (EDCs), among others [2,3,4,5]. The key actor of the HPG axis is the hypothalamic gonadotropin-releasing hormone (GnRH), a decapeptide able to induce the discharge of pituitary gonadotropins (the follicle-stimulating hormone (FSH) and luteinizing hormone (LH)) which in turn sustain the production of sex steroids by gonads and successful gametogenesis as a result [2]. In addition to the intricate endocrine communication routes and the related feedback mechanisms, paracrine and autocrine communications along the HPG axis ensure the production of high-quality gametes [6]. Several modulators exert their activity within the hypothalamus, regulating the activity of GnRH-secreting neurons in response to exogenous and endogenous environmental “cues”; similarly, peripherally produced modulators directly affect gametogenesis with effects on reproduction and fertility. In this respect, the list of centrally and peripherally produced modulators of reproduction is growing and the knowledge of their related molecular and epigenetic signalling pathways is functioning to preserve reproduction and fertility, and to develop successful therapeutic strategies as well. The first issue of this Special Issue, focused on the “Central and Local Modulators of Reproduction and Fertility”, collects a total of 13 articles, 6 review articles and 7 research articles, and expands the current knowledge on central and local modulators of reproduction and fertility in both physiological and pathological conditions. 

The first section of this Special Issue includes five basic review articles focused on gonadal sex determination and the role of methylation in reproduction, the circadian clock and corpus luteum.

During embryo development, gonadal sex determination commits a bi-potential undifferentiated gonad toward a “male” or “female” fate with the formation of an ovary or testis (differentiated gonads). This process requires cell proliferation, differentiation, migration, cell-specific interactions and gene expression and is under the control of well-defined molecular pathways. Cell adhesion molecules guarantee the structure and the integrity of the developing and adult gonads. In this respect, the review article by Piprek et al. [7] is focused on the role of the main cadherins (i.e., E- and N-cadherin) in germline and gonad development, male and female gametogenesis, reproduction and fertility. The manuscript provides a comprehensive overview on the known molecular mechanisms, but also suggests several missing points to be further investigated in the complex interaction between germinal and somatic cells.

The oestrogen-related signalling pathways in the developing gonads have been reviewed by Stewart et al. [8] in physiological condition and in the presence of exogenous oestrogens. Non-genomic and genomic pathways of oestrogen signalling, and molecular mechanisms occurring through the alteration of mitogen-activated protein kinase and SRY-box transcription factor 9 in somatic cells have been reported; both are responsible for cell fate decision, the suppression of testis genes and the activation of ovarian genes. This issue deserves particular interest due to the large presence in the environment of EDCs with oestrogenic activity and their possible impact on human reproductive health [3].

Methylation is a well-known biochemical process in which methyl groups, -CH_3_, are covalently bound to a large number of substrates. The addition of methyl groups to CpC islands in DNA or histone protein tails represents the major molecular mechanism in imprinting and epigenetic modulation of gene expression, and is capable of affecting embryo development, embryo health and disease load in the adult life [9,10]. The review article by Menezo et al. [11] is focused on the methylation process occurring during gametogenesis, early and late embryo development in physiological conditions and following the environmental exposure to EDCs. 

Several biological functions, included hormonal secretion, require the activation of a circadian clock system. Lifestyle, particularly stress, can interfere in the expression of “clock genes”, thus affecting the activity of the HPG axis and causing, in turn, poor reproductive outcomes. The review article by Sciarra et al. [12] provides a comprehensive overview on the complex interactions among circadian rhythms, hormones and fertility in both animal models and humans. At molecular levels, the recent findings on the complex network between the clock machinery, which includes several clock-specific genes, and reproductive hormones have been reviewed. 

The last review article is focused on corpus luteum, a transient endocrine structure in the ovary that serves as the primary source of progesterone during the menstrual/oestrous cycle and early pregnancy. In their review, Przygrodzka et al. [13] summarises the LH-dependent mechanisms that drive the fate of the corpus luteum and its ability to produce steroids. Well-orchestrated communications among mitochondria, endoplasmic reticulum, lipid droplets, cytoskeleton, lysosome and autophagosomes occur in luteal cells in response to LH signalling in order to modulate autophagy, stabilise mitochondria, activate lipolysis and stimulate the mobilisation of cholesterol to control autophagy and steroidogenesis in turn. 

The second section of this Special Issue collects four research articles focused on the central and local modulators of reproduction and fertility in vertebrates, from fish to humans. In this respect, the use of different experimental models has a recognised role to elucidate the evolutionarily conserved master signalling system in endocrinology [14].

In fish, glucocorticoids are directly involved in the endocrine control of reproduction, with basal levels resulting in positive modulators and higher levels resulting in negative effectors in reproduction. Hence, Maradonna et al. [15] used a multidisciplinary approach that included the analysis of tissue composition by Fourier transform infrared imaging to investigate the effects of glucocorticoid receptor knockout (*gr^−/−^*) in zebrafish brain, liver and ovary. Although significant effects were not observed in the liver, the kisspeptin system—the main gatekeeper of GnRH neurons in mammals [16]—was impaired in the brain of *gr^−/−^* mutant fish; in the ovary, the expression rate of key genes involved in oocyte maturation and ovulation were altered in the mutant animals that displayed reduced ovulation and fertility rate with respect to *wild type* females. Furthermore, altered oocyte composition and differences in the molecular structure of the zona radiata layer of *gr^−/−^* follicles were also observed, confirming the need of glucocorticoid signalling in fish reproduction, at least. 

Melatonin, the secretion product of the pineal gland, affects the HPG axis at multiple levels, but its activity on the hypothalamic GnRH-secreting neurons is not fully understood. Rijal et al. [17] measured melatonin levels in prepubertal and adult mice, revealing higher circulating melatonin levels in prepubertal animals and suggesting a melatonin-dependent inhibitory effect on GnRH production before puberty. Then, they carried out single-cell electrophysiological studies to investigate the direct activity of melatonin on GnRH-secreting neurons, revealing a new modulatory network in which melatonin suppressed the kainate receptor mediated-excitatory activity on the GnRH neurons in male and female prepubertal mice [17]. 

Well-known, centrally produced modulators of the HPG axis are produced also at periphery within the gonads and in reproductive tissues [2,6].

This is the case of kisspeptins, the cleavage product of the Kiss1 precursor that is able to bind to the membrane G-coupled-receptor named Kiss1R [16]. In spite of the recognised role of Kiss1/Kiss1R within the hypothalamus as positive modulators of GnRH secretion, both the ligands and receptor are produced within the gonads, reproductive tissues and gametes. Nevertheless, in the males, the need for intragonadal kisspeptin signalling to gain successful spermatogenesis is still debated [18,19]. Hence, Gloria et al. [20] used the features of a dog, an animal model close to humans, to characterise the kisspeptin system in the testis and spermatozoa collected at different tracts of the epididymis. The study revealed the presence of Kiss1/Kiss1R in dog testes, whereas Kiss1R only was detected in spermatozoa. The reported increase of Kiss1R on the sperm surface alongside the maturation during epididymal transit and the presence of Kiss1 in epididymal fluid suggests a possible new functional role of the system in sperm maturation and storage, as also postulated by recent reports in a rat model [21].

Sirtuin1 (Sirt1) is a nicotinamide adenosine dinucleotide (NAD^+^)-dependent deacetylase that removes acetyl groups from protein substrates, including histone proteins and transcriptional factors or cofactors. As a consequence, Sirt1 is a well-known “longevity factor” involved in ageing, cell proliferation, differentiation or death through the epigenetic modulation of gene expression in several species [4]. Sirt1 also has the ability to regulate the activity of the HPG axis in response to nutritional status [22], and its knockout causes male infertility only since the protein participates in several key stages of spermatogenesis and sperm maturation [23]. Hence, there is a need for additional studies in the intratesticular characterisation of this deacethylase. The research article by Wahab et al. [24] promptly analyses the expression of Sirt1 in the testes of primates and non-primate species during the annual reproductive cycle, revealing high Sirt1 protein levels in germ cells at mitotic, meiotic and post-meiotic phase. Interestingly, for the first time in primates, colocalisation of Sirt1 with the myokine irisin in primate spermatogonia and the irisin-dependent expression of *Sirt1* mRNA have been reported in primate spermatogonia.

The third section of the Special Issue contains one review article and two research articles focused on the toxic effects of environmental pollutants in reproduction. In recent years, this topic became hot due to the large use of environmental pollutants, particularly plastics and plasticisers that interfere in the endocrine system due to their ability to agonise or antagonise specific hormonal-mediated signalling pathways [3,25,26,27]. EDCs can contaminate air, soil and water, can enter the food chain and can exert toxic effects on reproduction following exposure through inhalation, ingestion or absorption. Effects on male and female reproduction depend on doses, exposure routes, exposure time and life stage, with exposure at gestation, neonatal and juvenile phases more deleterious than in adulthood [3,27]. Furthermore, the discovery that EDCs interfere in the reproductive functions of the exposed organism and can epigenetically affect the health status of the offspring recently emerged [3,5,27], suggesting the need for additional studies in the field and the development of safe alternatives to preserve reproduction, fertility and health.

In such a context, the review article by Plunk and Richards [28] discusses the current findings on EDCs that can be inhaled (i.e., brominated and organophosphate flame retardants, diesel exhaust, polycyclic aromatic hydrocarbons, cadmium and lead, anthropogenic halogenated dioxin 2,3,7,8-tetrachlorodibenzo-p-dioxin (TCDD) and polychlorinated biphenyls), revealing their possible deleterious impact on the physiology of the HPG axis and fertility in both sexes. 

The research article by Hunter et al. [29] analysed the effects of in utero and neonatal exposure to the xenobiotics Dibutyl Phthalate (DBP) and Diethylstilbestrol (DES) on hypothalamic gene expression and behaviour in the offspring. The authors demonstrate that DES treatment led to significant changes in hypothalamic gene expression and behaviour [29], thus confirming that impacting brain development at critical times can have long-term effects on health. 

The effects of the plasticiser Bisphenol A (BPA) on Sertoli cells, the nurturing cells that in the testis protect and allocate germ cells within germinal epithelium, were investigated by Rossi et al. [30]. In their manuscript, the authors tested a non-cytotoxic dose of BPA in mouse primary Sertoli cells, revealing significant effects on the expression rate (mRNA or protein) of the endocannabinoid system. This signalling system is one of the major central and local modulators of reproduction and fertility [31,32] and comprises ligands, receptors, biosynthetic and hydrolysing enzymes. Interestingly, in their manuscript Rossi et al. demonstrated that the BPA effects on the production of inhibin B occurred through the signalling of cannabinoid receptor CB2 and the vanilloid receptor TRPV1, thus including endocannabinoid signalling in the list of BPA intratesticular targets [30]. 

The last section of this Special Issue contains one research article reporting data potentially useful in clinical practice. This pivotal study by Notarstefano et al. [33] compares the effects of three controlled ovarian stimulation (COS) protocols (i.e., urinary FSH, recombinant FSH, or human menopausal gonadotropin) on the metabolic state and endocannabinoid system of cumulus cells from 42 normal-responder women. This pivotal study reveals that each COS protocol causes specific changes in metabolism, composition and the endocannabinoid system of cumulus cells. If confirmed in a larger cohort of patients, this finding could become relevant for the selection of the appropriate COS protocol in clinical practice. 

In conclusion, despite experimental evidence, modulators and regulatory mechanisms in reproduction and fertility yet remain to be fully elucidated; hence, there is a need to fill this gap in order to preserve reproduction and devise clinically effective treatment strategies in infertility. The manuscripts included in this Special Issue of the *IJMS* surely add insights and new perspectives to the field. 
